# Geochemical Responses to Anthropogenic and Natural Influences in Ebinur Lake Sediments of Arid Northwest China

**DOI:** 10.1371/journal.pone.0155819

**Published:** 2016-05-13

**Authors:** Long Ma, Jinglu Wu, Jilili Abuduwaili, Wen Liu

**Affiliations:** 1 State Key Laboratory of Desert and Oasis Ecology, Xinjiang Institute of Ecology and Geography, Chinese Academy of Sciences, Urumqi, China; 2 State Key Laboratory of Lake Science and Environment, Nanjing Institute of Geography and Limnology, Chinese Academy of Sciences, Nanjing, China; 3 CAS Research Center for Ecology and Environment of Central Asia, Urumqi, China; Institute of Tibetan Plateau Research, CHINA

## Abstract

Geochemical concentrations were extracted for a short sediment core from Ebinur Lake, located in arid northwest China, and mathematical methods were used to demonstrate the complex pattern of the geochemical anomalies resulting from the temporal changes in natural and anthropogenic forces on the lake sediments. The first element assemblage (C1) (aluminum, potassium, iron, magnesium, beryllium, etc.) was predominantly terrigenous; among the assemblage, total phosphorus and titanium were generally consistent with aluminum except with regards to their surface sequences, which inferred the differences of source regions for terrigenous detrital material led to this change around ca. 2000AD. The second assemblage (C2) (calcium and strontium) was found to have a negative relationship with aluminum through a cluster analysis. The third assemblage (C3) included sodium and magnesium, which were influenced by the underwater lake environment and deposited in the Ebinur depression. The concentration ratio of C1/(C1+C2) was used as an indicator for denudation amount of detrital materials, which was supported by the values of magnetic susceptibility. The enrichment factors for heavy metals suggested that the influence of human activities on heavy-metal enrichment in Ebinur Lake region was not severe over the past century. Prior to the 1960s, geochemical indicators suggested a stable lacustrine environment with higher water levels. Beginning in the 1960s, high agricultural water demand resulted in rapid declines in lake water level, with subsequent increases of lake water salinity, as evidenced by enhanced sodium concentration in lake core sediments. During this period, anthropogenic activity also enhanced the intensity of weathering and the denudation of the Ebinur watershed.

## Introduction

Geochemical elements in lake sediments can be influenced by both natural and anthropogenic processes, and these element assemblages can be used to investigate the contributions of different forcing mechanisms to changes in sedimentary environments [1, 2]. Based on studies examining long-term paleo-environmental changes in lacustrine environments, natural processes affect the transportation of geochemical elements from watersheds to lakes [3–5]. Over the past several decades, human activities have accelerated cycling of geochemical elements and resulted in elevated metal deliveries to water bodies [6–8]. Lacustrine sediments provide historic records of natural evolution and anthropogenic influences on lakes and their watersheds [9–12].

As part of the elemental composition of lake sediments, heavy metals are potentially toxic to ecological systems through the processes of bio-accumulation and bio-magnification [13–15]. It is therefore important to understand how climatic variations and anthropogenic activities influence the concentrations of geochemical constituents, especially heavy metals. Sediment cores can provide chronologies of metal concentrations in sedimentary sequences, and have been used to reveal human influences on heavy metal accumulation. However, the history of contamination in the arid environments of socially developing regions, particularly northwest China, has not been widely studied in comparison to developed regions.

Ebinur Lake is a closed lake situated in arid northwest China, which is sensitive to climatic and environmental changes [16]. Using lake sediment cores, our goals are 1) to determine the concentrations of geochemical constituents that characterized the Ebinur Lake sediments prior to human activities, 2) to evaluate the enrichment trend of heavy metals and the degree of heavy-metal accumulation, and 3) to determine the possible geochemical sources that influenced metal concentrations in the Ebinur Lake sediments.

### Study area

**Fig 1:**
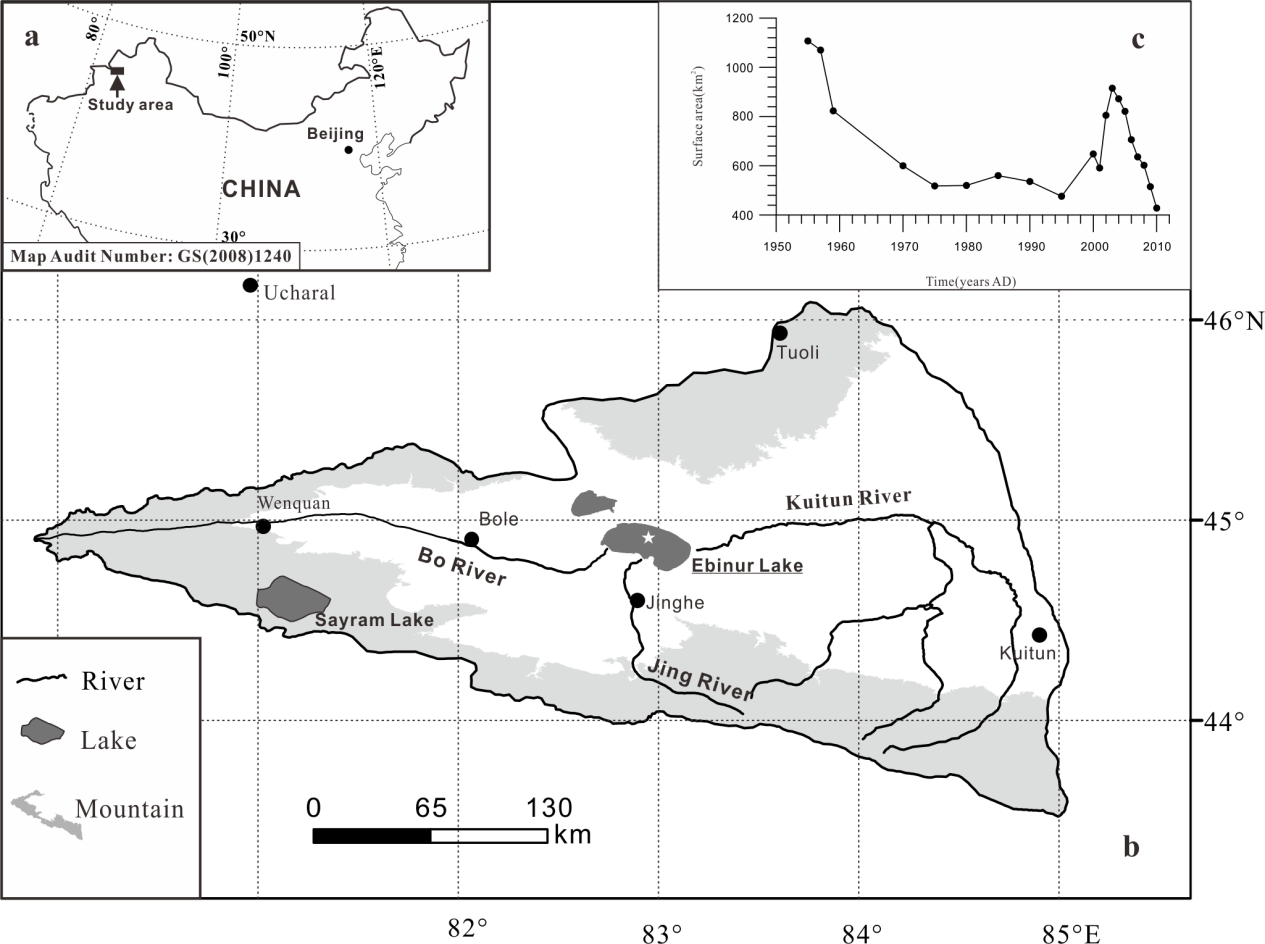
Sketch map of the geographic location of (a) the watershed of Ebinur Lake, (b) the sampling site, and (c) the Ebinur lake variation.

Ebinur Lake is a shallow, closed lake in northwest China (Fig 1), which belongs to the Ebinur Lake Wetland National Nature Reserve. It is also a rift lake, formed in the Himalayan orogeny, and deposited thick Quaternary unconsolidated sediments [17]. The lake drainage area is 50,321 km^2^, including 24,317 km^2^ of mountainous terrain. Ala Mountain borders the lake in the north and northwest, Boer Tala Valley is to the west; the Jing River pluvial fan is to the south, and sand dunes around the Kuitun River are to the east [16]. This lake receives surface runoff from the Boertala, Jing and Kuitun Rivers. The depth of the lake water averages 1.2 m, with a maximum depth of 3.5 m. Total dissolved solids in the lake range between 85 and 124 g/L^-1^. As part of a field investigation from 1987–1989 [18], Lake Ebinur was hydrochemically classified as a sulphate-sodium-II type lake. Based on a field survey conducted in 2009, it was shown that the major ions in Lake Ebinur were chlorine and sodium, and the hydrochemical classification subsequently changed from sulphate-sodium-II type to chloride-sodium-II type [19]. The annual total precipitation in Ebinur Lake watershed is approximately 95 mm, whereas annual evaporation totals for the watershed can reach 1315 mm [16]. During the past half century, the economy in Ebinur Lake drainage has made rapid progress. It is an agriculture-based economy, and the small-scale enterprise becomes the main types of the secondary industry sector. From the economic data of Xinjiang economic statistics yearbooks [16], gross domestic product (GDP) in Jinghe county (Fig 1) rose from about $ 410 000 in 1955 to $ 540 million in 2010. The primary industry increased from about $ 350 000 in 1955 to $ 300 million in 2010, and the secondary industry rose from about $10 000 in 1955 to $ 85 million in 2010. In the agriculture department, total sown area of farm crops enlarged from 5 720 ha in 1955 to 10^3^ ha in 2010. The variation in the surface area of the lake is shown in Fig 1. The surface area of this lake was about 1070 km^2^ in 1950, and then, experienced a rapid contraction. In 1972, the lake area decreased to 589 km^2^. In the late 1990s, Ebinur Lake started to expand; however, the lake area shrank sharply from 2004. The variation of Lake Ebinur area is jointly controlled by human activities and climate change. However, the human activity was mainly responsible for Ebinur Lake shrinking quickly over the past half century [16].

## Materials and Methods

In July 2011, with the support and permission of the Administrative Bureau of Ebinur Lake Wetland National Nature Reserve, a short sedimentary core (50-cm long) was obtained at the site (82.9603°E, 44.9113°N) with water depth of 2.4 m using a piston-percussion corer with a 60 mm inside-diameter Perspex tube. After collection, the core was vertically maintained and immediately sub-sampled at intervals of 1 cm. The age-depth relationship was determined by the dating of ^210^Pb and ^137^Cs, which were analyzed using an EG&G Ortec Gamma Spectrometer.

Bulk sediments in ~0.125 g were dried at 105°C, ground to 200-μ mesh size, and then, they were totally digested with HF-HNO_3_-HClO_4_ in a Berghof MWS-3 microwave digester, and prepared for the determination of elements with a Leeman Labs Profile Inductively Coupled Plasma Spectrometer (ICP-AES). The relative error was determined to be less than 5%. National Standard Reference material (GBW0731) was used to determine major and minor element standards. Magnetic susceptibility (MS) was measured using a Bartington MS2 susceptibility meter (Bartington Instruments, Oxford, England). Samples were pretreated with 10–20 ml of 30% H_2_O_2_ to remove organic matter and with 10 ml of 10% HCl to remove carbonates, prior to particle size analysis. Particle size was determined with a Malvern Mastersizer 2000 analyzer with a measurement range of 0.02–2000 μm. The Mastersizer 2000 automatically determines size fractions with a measurement precision <1%. Carbonate content was determined by the volumetric calcimeter method [20], and the error in carbonate measurement was less than 5%. The above analyses were conducted in the State Key Laboratory of Lake Science and Environment, Chinese Academy of Sciences.

Correlation analysis was used to analyze the interrelationship and to assess geochemical associations among the major and trace elements [21]. Hierarchical cluster analysis (HCA) was used to reveal the similarity/dissimilarity between variables [22, 23]. The element concentrations were standardized using z-score transformation, which converts all elements to a common scale. Z-scores can be calculated from the following formula, z = (X - μ) / σ, where z is the z-score, X is the value of the element, μ is the mean value, and σ is the standard deviation [24]. Pearson correlation distance was calculated and the weighted pair-group method using centroid approach (median linkage) was selected for cluster analysis. The above statistical analyses were conducted using the SPSS 15.0 for Windows.

Enrichment factors (EFs) were calculated as the elemental content to background content ratio [6, 25]. In this work, background content was quantified by determining the average content in the bottom 10 cm of the core sample. Aluminum (Al) is usually chosen as a reference element to distinguish from anthropogenically introduced elements [21, 26]. This is because aluminum is typically of terrigenous origin, and is geochemically stable in supergene environments [27, 28]. Therefore, the EFs of the geochemical element were calculated using the equation, EF_s_ = (C_GE_/C_Al_)/(B_GE_/B_Al_), where C_GE_ was the elemental concentration in the core sediment; C_Al_ was the aluminum content in the core sediment; B_GE_ was the elemental concentration of natural background, and the B_Al_ was the aluminum content in the natural background.

## Results

**Fig 2:**
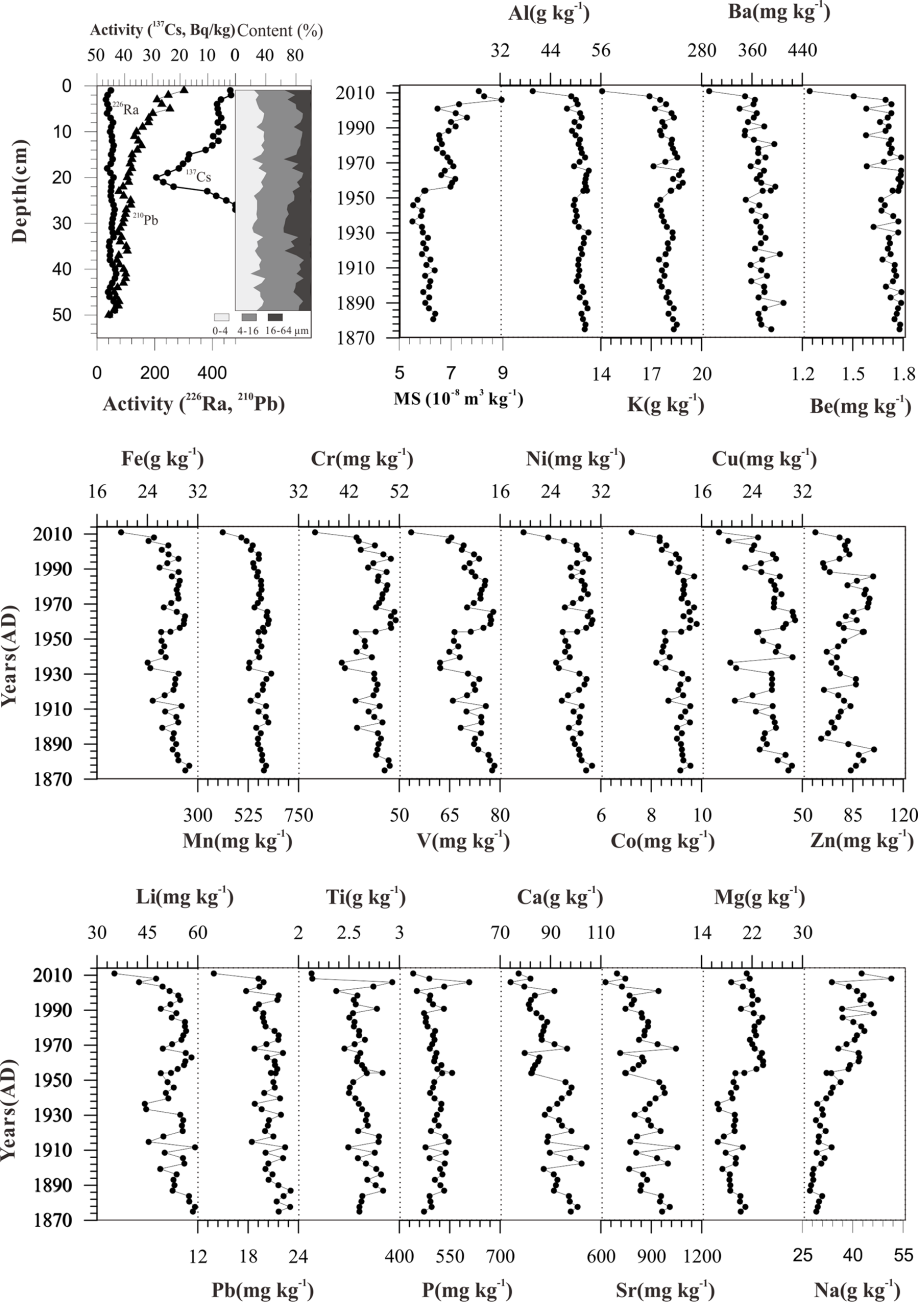
The specific activity of ^137^Cs, ^226^Ra and total ^210^Pb activity in Ebinur Lake core sediment and the profiles for elemental concentrations in Ebinur Lake core sediment.

The vertical distribution of ^210^Pb and ^137^Cs is shown in Fig 2. Based on the study findings, ^137^Cs accumulation began at 25 cm depth with specific activity value of zero in 1954, with peak concentrations measured at 20 cm depth in 1963, corresponding to the fallout maximum from nuclear weapons testing at this time [29, 30]. The unsupported ^210^Pb activity (^210^Pb_ex_) decreased to zero at close to 49 cm depth (Fig 2). A constant rate of supply model [30] was used to calculate the date of samples, which was consistent with the ^137^Cs chronology.

**Fig 3:**
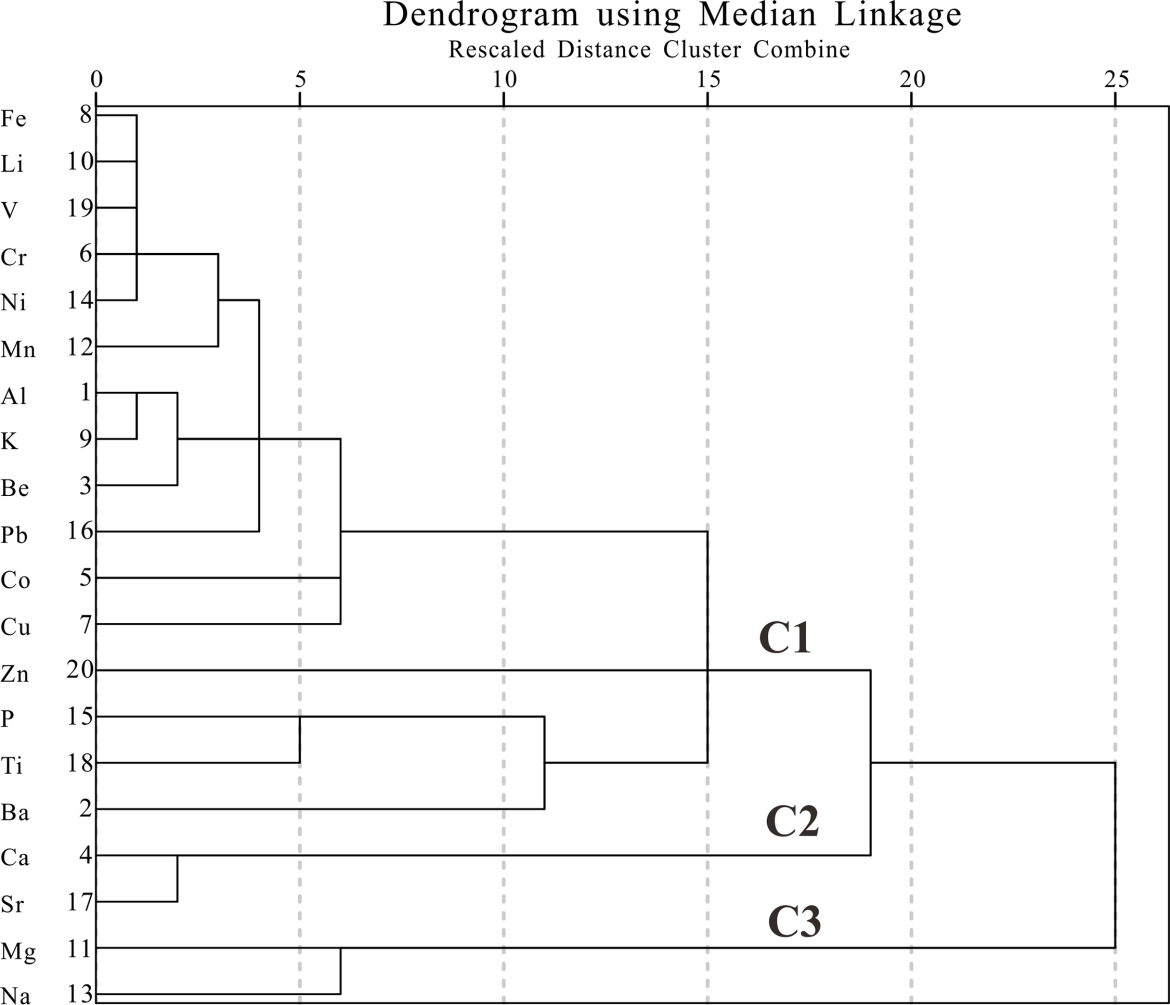
Hierarchical clustering of geochemical elements in the Ebinur Lake sediment core.

The average contents of the fractions of <4 μm, 4–16 μm, 16–64 μm and > 64μm are 31%, 47%, 21% and 1%, respectively (Fig 2). The Ebinur Lake core sediments are composed of relatively uniform fine-grained materials. The elemental content in Ebinur Lake sediments are presented in S1 Table and Fig 2. The HCA dendrogram categorizes these elements into several branches (Fig 3). The elements within a sub-cluster have more similarity than those not in sub-clusters. The results show that: (1) most elements, including Al, potassium (K), beryllium (Be), barium (Ba), titanium (Ti), sodium (Na), magnesium (Mg), zinc (Zn), vanadium (V), lead (Pb), cobalt (Co), chromium (Cr), nickel (Ni) and iron (Fe), had a similar distribution pattern which generally increased with depth. The observed time evolution of the zonal anomalies illustrates the apparently increase from the 1950s onwards. (2) Na and Mg had a negative correlation with Al. (3) In general, calcium (Ca) and strontium (Sr) also increased with depth, similar to Al; however, the wave direction of Ca and Sr was opposite to that of Al. (4) The trends of total phosphorus (P) and Ti were similar to Al; however at the sediment surface the trend of these elements was inconsistent with that of Al.

## Discussion

Various factors influenced the distribution and accumulation of geochemical elements, including sedimentary texture, mineral composition, oxidation/reduction state, adsorption/desorption processes and physical transportation [13, 31–33]. Profiles of representative elements are generally used to characterize the depositional environment [34, 35].

**Fig 4:**
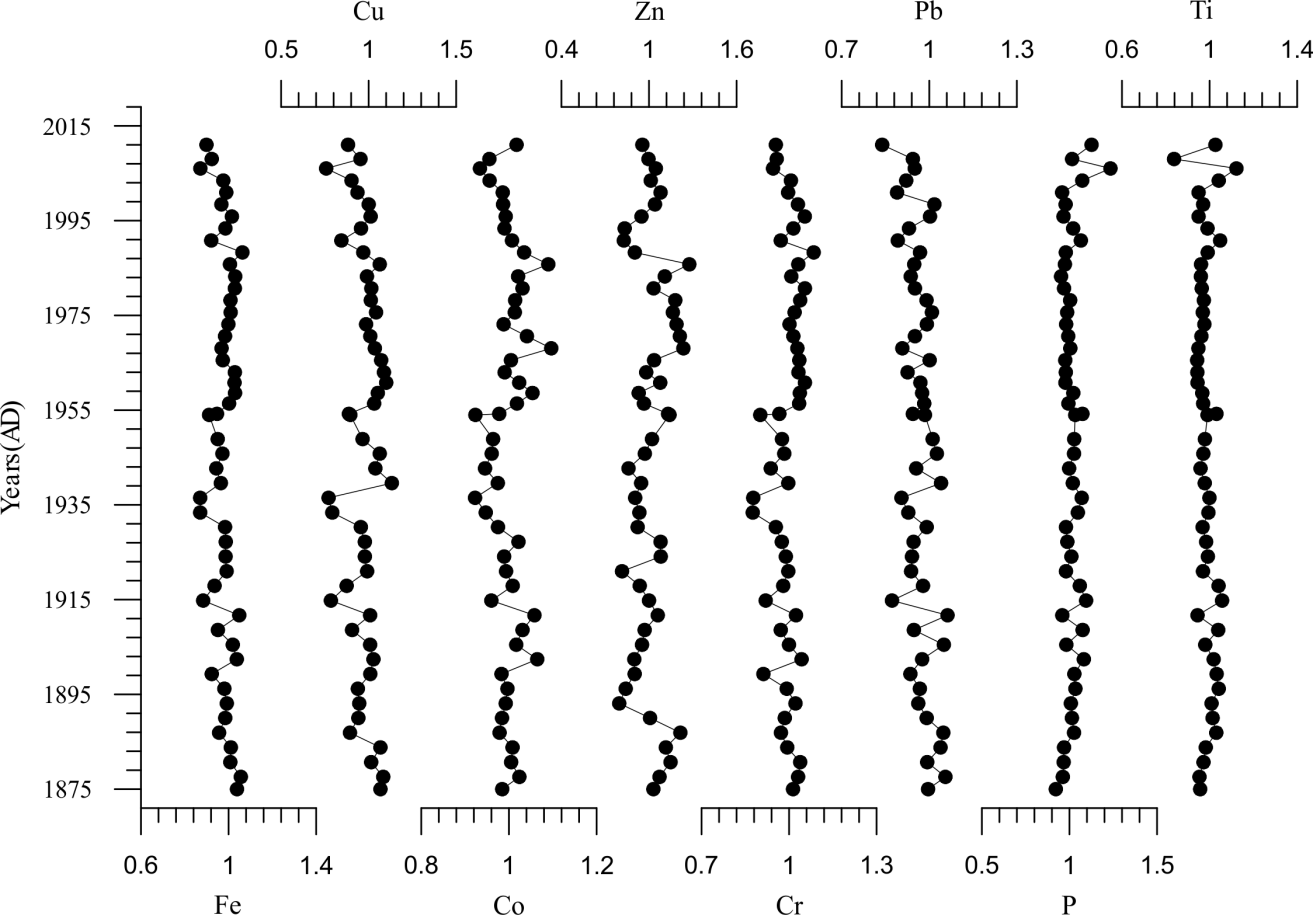
Representative enrichment factors for major and trace elements in the Ebinur Lake sediment core.

From the dendrogram of HCA (Fig 3), we found three primary element assemblages. The first element assemblage (C1) included Al, K, Fe, Mg, Be, etc. The positive relationships among the elements Al, K, Mg, Fe, and manganese (Mn) were observed with relatively high correlation coefficient (S2 Table), which suggests the same origin for them. Aluminum is extremely immobile, usually held in the lattice of aluminosilicate minerals and regarded as a typical lithogenic element [36]. Al and K are major constituents of common silicate minerals and originated from the weathering release of parent materials in the local bedrock [37]. The EFs for heavy metals and P were used to identify and quantify anthropogenic interference; however, the EFs showed inconspicuous variations with a mean value of approximately one (Fig 4). This suggests that the influences of human activity on the enrichments of heavy metals and P in this region was not severe over the past century. It must be noted, however, that P and Ti were strongly correlated (r = 0.811, p<0.01) (S2 Table). From the Fig 4, from ca. 2000 AD, we observed a relatively significant variation for the EFs of Ti and P, however, the EFs for both of them didn’t exceed to 1.24. The results inferred the differences of source regions for terrigenous detrital material led to this change around ca. 2000AD.

The second assemblage (C2, Fig 3) (Ca and Sr) was found to have a strong positive relationship (r = 0.928, p<0.001). Due to similar ionic radii (r_Sr_ = 0.113 nm vs. r_Ca_ = 0.099 nm) and charge [38], the element assemblage of Ca and Sr is believed to behave similarly with each other, with Sr typically found in calcium-bearing minerals such as plagioclase [39]. On the whole, all the elements in first and second assemblages had downward trends, which were opposite to the ones in third group, but no significant correlation was present between the first and second assemblages. However, after conduct a detrend analysis with subtracting the trend estimated from the linear regression model, there was a strong negative correlation between Ca and Al (r = -0.592, p<0.001), suggesting a dissolved terrigenous bicarbonate input, which was deposited in the lake as solid carbonate.

**Fig 5:**
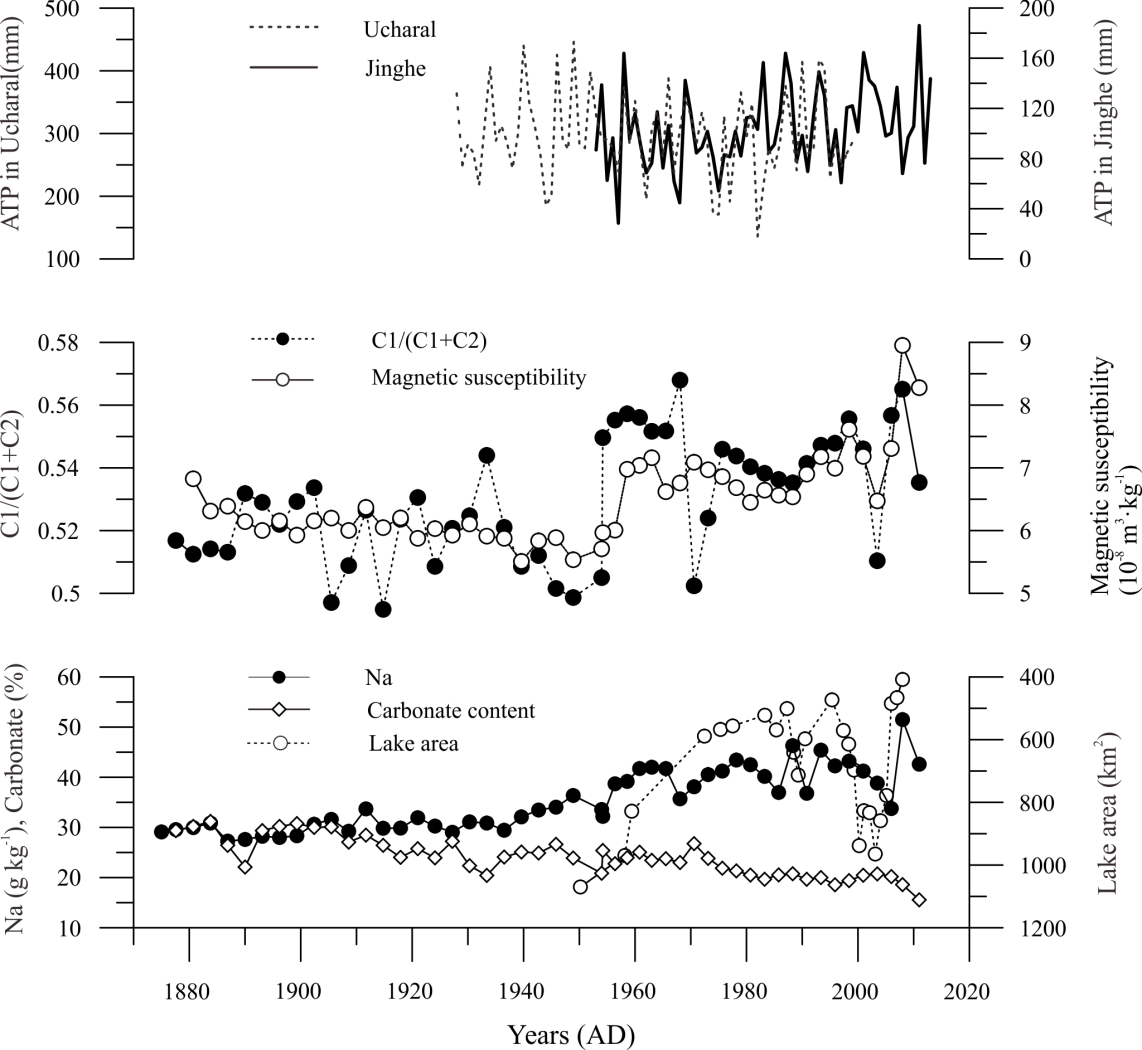
Geochemical evolution of the Ebinur Lake sediment core compared with the lake surface area change with axis scale in reverse, and meteorological data in the surrounding area.

The third element assemblage (Na and Mg) consisted of autogenetic evaporative minerals that were influenced by the lacustrine environment and deposited in the Ebinur depression. In the process of arid closed lake evolution, the dominated sedimentary minerals were following the carbonates–sulfates—chlorides sequence [40]. In the earlier evolutionary stage, Calcium-magnesium carbonates reign supreme over the mineral sedimentation. Meanwhile, natron (Na_2_CO_3_•10H_2_O) and trona(Na_2_CO_3_•NaHCO_3_•2H_2_O) will be deposited with a small amount. In the latter period, the lake water salinity increases as the depth decreased, sulfates (e.g. Na_2_SO_4_•10H_2_O, Na_2_SO_4_) with greater solubility will be precipitate before halite can form [40]. From the carbonate content of Ebinur Lake sediments (Fig 5), although it shows a trend of temporal decrease, the carbonate content in lake sediment fluctuated in a narrow range, which indicated that Ebinur Lake had already undergone the carbonate evolutionary stage since 1880 AD. In recent years, the shrinking trend of Ebinur Lake exacerbated. Sodium can be considered as a water level indicator. Subsequently, as lake levels dropped, salinity concentrations measured in the sediment core increased. A mass of sodium salt precipitated from aqueous solution, which induced the concentration of Na had the opposite trend with the lake level. The third assemblage (C3) included sodium and magnesium, were influenced by the underwater lake environment and deposited in the Ebinur depression.

Fig 5 shows the climatic data around the Ebinur Lake. The data of annual total precipitation (ATP) from 1955–2013 at Jinghe weather station (location showed in Fig 1) were provided by the National Meteorological Administration of China (http://data.cma.cn), and the ATP data at Ucharal station (E 80.93°, N 46.17°) are from the reference [41]. There are no sudden changes around 1960s, which inferred that the climate variation was not enough to induce the significant change in geochemical compositions of Ebinur Lake core sediments. Corresponding to local economic development, an increase in agricultural water demand began in the 1960s, the Ebinur Lake experienced a rapid contraction due to the sustained increase of irrigation water requirements and decreased annual precipitations. In the late 1990s, Ebinur Lake area expanded due to the reduced agricultural water consumption with increased annual precipitations, and the lake area peaks to 903 km^2^ in 2003[42, 43]. Nevertheless, the Ebinur Lake shrank sharply from 2004 with the combination of reduced rainfall and increased farmland under irrigation [44]. Prior to the 1960s, Na was present at relatively constant lower values, suggesting a stable lacustrine environment with higher water levels. With lake surface area experiencing a constantly change, the Na concentration in lake sediments varied accordingly (Fig 5).

Following the above-mentioned discussion, the first element assemblage (C1) was predominantly terrigenous clastic. The climate has the important influence on the chemical weathering process. Due to the extreme arid climate in Ebinur watershed with weak chemical weathering, the Al come from the earth surface are not in free state. Among these elements in the first assemblage, the K [45], Al [46], and Fe [47] have been commonly chosen as conservative lithogenic elements due to their immobile behavior during weathering and pedogenesis. For the dry climate dominated on the Ebinur Lake watershed, the earth surface materials experience the incipient stage of chemical weathering characterized by leaching of Ca and Na, and not reached the secondary process characterized by the removal of K [48]. The second assemblage (C2) was from dissolved terrigenous bicarbonate inputs, which was deposited in the lake as solid carbonate. And then, the concentration ratio of C1/(C1+C2) can be used as an indicator for denudation amount of detrital materials. Meanwhile, the interpretation for our ratio’s environmental meaning was supported by the values of magnetic susceptibility (Fig 5). Magnetic minerals in lake sediments mainly come from surface materials of lake watershed. Land reclamation and vegetation deterioration will increase surface erosion. Higher values of magnetic susceptibility over the past few decades indicate greater erosion in the lake drainage basin resulting from human activity [49]. In addition to falling lake water levels [16], eroded material was transported via fluvial processes and deposited in the lake, resulting in further intensification of clastic deposits. In general, the anthropogenic factors have influence on geochemical composition of lake sediments through the means of strengthening the lake area change and surface erosion.

## Conclusions

Using enrichment factors and multivariate statistical techniques, geochemical responses of Ebinur Lake sediments to anthropogenic and natural influences were determined. Most elements (including Al, K, Fe, Mg and Be) originated mainly from terrigenous detrital inputs. Ca and Sr originated mainly from terrigenous bicarbonate inputs and were deposited in the lake as solid carbonate. Na and Mg are autogenetic evaporative minerals that were influenced by the lacustrine environment and deposited in the Ebinur depression. The EFs for heavy metals and total phosphorus showed inconspicuous variations with a mean value of approximately one, which suggested that the influences of human activity on heavy metal accumulation in this region were not severe over the past century.

Since the 1960s, agricultural water demand has increased considerably, and a rapid decrease in the lake water level was inferred from the higher sodium concentrations in the sediments closest to the surface. With an increase in the surface erosion rate due to human activities, fluvial activity deposited a considerable amount of eroded material in the lake, which increased the relative content of clastic deposits.

Overall, the intensity of human activity in this region was not severe over the past century, which referred to the influences on heavy metal enrichment. The human activities had the deep impacts on geochemical composition of lake sediments through the means of influencing the lake area change and surface erosion.

## Supporting Information

S1 Table The geochemical data of elemental compositions in Ebinur Lake sediments. (PDF)

S2 Table Pearson correlation matrix for element concentration in Ebinur Lake sediments. (PDF)

## References

[1] Koinig KA, Shotyk W, Lotter AF, Ohlendorf C, Sturm M. 9000 years of geochemical evolution of lithogenic major and trace elements in the sediment of an alpine lake–the role of climate, vegetation, and land-use history. J Paleolimnol. 2003;30(3):307–320.

[2] Routh J, Meyers PA, Gustafsson Ö, Baskaran M, Hallberg R, Schöldström A. Sedimentary geochemical record of human–induced environmental changes in the Lake Brunnsviken watershed, Sweden. Limnol Oceanogr. 2004;49(5):1560–1569.

[3] Braun M, Hubay K, Magyari E, Veres D, Papp I, Bálint M. Using linear discriminant analysis (LDA) of bulk lake sediment geochemical data to reconstruct lateglacial climate changes in the South Carpathian Mountains. Quat Int. 2013;293:114–122.

[4] Kylander ME, Klaminder J, Wohlfarth B, Löwemark L. Geochemical responses to paleoclimatic changes in southern Sweden since the late glacial: the Hässeldala Port lake sediment record. J Paleolimnol. 2013;50(1):57–70.

[5] Biskaborn BK, Herzschuh U, Bolshiyanov D, Savelieva L, Diekmann B. Environmental variability in northeastern Siberia during the last ~ 13,300 yr inferred from lake diatoms and sediment–geochemical parameters. Palaeogeogr Palaeoclimatol Palaeoecol. 2012;329–330:22–36.

[6] Zahra A, Hashmi MZ, Malik RN, Ahmed Z. Enrichment and geo-accumulation of heavy metals and risk assessment of sediments of the Kurang Nallah—feeding tributary of the Rawal Lake Reservoir, Pakistan. Sci Total Environ. 2014;470:925–933. 10.1016/j.scitotenv.2013.10.017 24239813

[7] Li S, Hu X, Tang Y, Huang C, Xiao W. Changes in lacustrine environment due to anthropogenic activities over 240 years in Jiuzhaigou National Nature Reserve, southwest China. Quat Int 2014;349:367–375.

[8] Zeng H, Wu J, Liu W. Two-century sedimentary record of heavy metal pollution from Lake Sayram: A deep mountain lake in central Tianshan, China. Quat Int. 2014;321:125–131.

[9] Leorri E, Mitra S, Irabien MJ, Zimmerman AR, Blake WH, Cearreta A. A 700year record of combustion-derived pollution in northern Spain: Tools to identify the Holocene/Anthropocene transition in coastal environments. Sci Total Environ. 2014;470:240–247. 10.1016/j.scitotenv.2013.09.064 24135492

[10] Gao C, Lin Q, Bao K, Zhao H, Zhang Z, Xing W, et al Historical variation and recent ecological risk of heavy metals in wetland sediments along Wusuli River, Northeast China. Environ Earth Sci. 2014;72(11):4345–4355.

[11] Boyle J, Chiverrell R, Schillereff D. Lacustrine archives of metals from mining and other industrial activities-a geochemical approach In: Blais JM, Rosen MR, Smol JP, editors. Environmental Contaminants. Netherlands: Springer; 2015 pp. 121–159.

[12] Guo W, Huo S, Ding W. Historical record of human impact in a lake of northern China: Magnetic susceptibility, nutrients, heavy metals and OCPs. Ecol Indic. 2015;57:74–81.

[13] Buccolieri A, Buccolieri G, Cardellicchio N, Dell'Atti A, Di Leo A, Maci A. Heavy metals in marine sediments of Taranto Gulf (Ionian Sea, southern Italy). Mar Chem. 2006;99(1):227–235.

[14] Ennouri R, Chouba L, Magni P, Kraiem M. Spatial distribution of trace metals (Cd, Pb, Hg, Cu, Zn, Fe and Mn) and oligo-elements (Mg, Ca, Na and K) in surface sediments of the Gulf of Tunis (Northern Tunisia). Environ Monit Assess. 2010;163(1–4):229–239. 10.1007/s10661-009-0829-5 19277885

[15] Hornberger MI, Luoma SN, van Geen A, Fuller C, Anima R. Historical trends of metals in the sediments of San Francisco Bay, California. Mar Chem. 1999;64(1):39–55.

[16] Ma L, Wu J, Liu W, Abuduwaili J. Distinguishing between anthropogenic and climatic impacts on lake size: a modeling approach using data from Ebinur Lake in arid northwest China. J Limnol. 2014;73(2): 148–155.

[17] Luo M, Yang L, Zhang S. Natural landscapes and strategies of regional sustainable development in Ebinur lake region in Xinjiang. Journal of Northeast Forestry University. 7(2): 16–19.

[18] Li T. Chemical evolution of Aibi lake water. J Lake Sci. 1993;5(3):234–243. Chinese.

[19] Wu J, Liu W, Zeng H, Ma L, Bai R. Water Quantity and Quality of Six Lakes in the Arid Xinjiang Region, NW China. Environ Proc. 2014;1(2):115–125.

[20] Loeppert RH, Suarez DL. Carbonate and Gypsum In: Sparks DL, editor. Methods of Soil Analysis. Part 3. Chemical Methods. Madison, WI, USA: Soil Science Society of America, American Society of Agronomy; 1996 pp. 457–604.

[21] Ma L, Wu J, Abuduwaili J. Geochemical evidence of the anthropogenic alteration of element composition in lacustrine sediments from Wuliangsu Lake, north China. Quat Int. 2013;306:107–113.

[22] Devic G, Djordjevic D, Sakan S. Natural and anthropogenic factors affecting the groundwater quality in Serbia. Sci Total Environ. 2014;468:933–942. 10.1016/j.scitotenv.2013.09.011 24080418

[23] Holmes E, Loo RL, Stamler J, Bictash M, Yap IK, Chan Q, et al Human metabolic phenotype diversity and its association with diet and blood pressure. Nature. 2008;453(7193):396–400. 10.1038/nature06882 18425110 PMC6556779

[24] Milligan GW, Cooper MC. A study of standardization of variables in cluster analysis. Journal of Classification. 5(2):181–204.

[25] Qi S, Leipe T, Rueckert P, Di Z, Harff J. Geochemical sources, deposition and enrichment of heavy metals in short sediment cores from the Pearl River Estuary, Southern China. J Mar Syst. 2010;82 (4 Suppl 1): S28–S42.

[26] Muller J, Wüst RA, Weiss D, Hu Y. Geochemical and stratigraphic evidence of environmental change at Lynch's Crater, Queensland, Australia. Glob Planet Change. 2006;53(4):269–277.

[27] Schropp SJ, Lewis FG, Windom HL, Ryan JD, Calder FD, Burney LC. Interpretation of metal concentrations in estuarine sediments of Florida using aluminum as a reference element. Estuaries. 1990;13(3):227–235.

[28] Reimann C, Caritat Pd. Intrinsic flaws of element enrichment factors (EFs) in environmental geochemistry. Environ Sci Tech. 2000;34(24):5084–5091.

[29] Pennington W, Tutin T, Cambray R, Fisher E. Observations on lake sediments using fallout 137Cs as a tracer. Nature. 1973; 242: 324–326. 4699049 10.1038/242324a0

[30] Appleby P. Chronostratigraphic techniques in recent sediments In: Last W, Smol J. editors. Tracking environmental change using lake sediments: Netherlands: Springer; 2001 pp. 171–203.

[31] Sagheer AA. Geochemistry in surface sediments of the Kwar Katib lagoon, Red sea, Yemen. J Environ Res Manag. 2013;4(4):242–248.

[32] Taghinia Hejabi A, Basavarajappa H, Qaid Saeed A. Heavy metal pollution in Kabini River sediments. Int J Environ Resour Res. 2010;4(4):629–636.

[33] Vrhovnik P, Šmuc NR, Dolenec T, Serafimovski T, Dolenec M. An evaluation of trace metal distribution and environmental risk in sediments from the Lake Kalimanci (FYR Macedonia). Environ earth Sci. 2013;70(2):761–775.

[34] Nameroff T, Balistrieri L, Murray J. Suboxic trace metal geochemistry in the eastern tropical North Pacific. Geochim Cosmochim Acta. 2002;66(7):1139–1158.

[35] Wu J, Ma L, Yu H, Zeng H, Liu W, Abuduwaili J. Sediment geochemical records of environmental change in Lake Wuliangsu, Yellow River Basin, north China. J Paleolimnol. 2013;50(2):245–255.

[36] Price N, Brand T, Pates JM, Mowbray S, Theocharis A, Civitarese G, Miserocchi S, Heussner S, Lindsay FS. Horizontal distributions of biogenic and lithogenic elements of suspended particulate matter in the Mediterranean Sea. Prog Oceanogr.1999;44:191–218.

[37] Wilcke W, Müller S, Kanchanakool N, Zech W. Urban soil contamination in Bangkok: heavy metal and aluminium partitioning in topsoils. Geoderma.1998;86:211–228.

[38] Dasch AA, Blum JD, Eagar C, Fahey TJ, Driscoll CT, Siccama TG. The relative uptake of Ca and Sr into tree foliage using a whole-watershed calcium addition. Biogeochemistry. 2006;80(1):21–41.

[39] Ekwere S. Li, F and Rb contents and Ba/Rb and Rb/Sr ratios as indicators of postmagmatic alteration and mineralization in the granitic rocks of the Banke and Ririwai Younger Granite complexes, Northern Nigeria. Mineralium Deposita. 1985;20(2):89–93.

[40] Einsele G. Continental Sediments In: Einsele G, editor. Sedimentary Basins: Evolution, Facies, and Sediment Budget. Berlin, Heidelberg: Springer Berlin Heidelberg; 2000 pp. 19–93.

[41] Williams MW, Konovalov VG. Central Asia Temperature and Precipitation Data, 1879–2003. USA National Snow and Ice Data Center. 2008; Available: 10.7265/N5NK3BZ8

[42] Ma L, Wu J, Abuduwaili J. The climatic and hydrological changes and environmental responses recorded in lake sediments of Xinjiang, China. J Arid Land. 2011; 3(1): 1–8.

[43] Ma M, Wang X, Veroustraete F, Dong L. Change in area of Ebinur Lake during the 1998–2005 period. Int J Remote Sens. 2007;28(24):5523–5533.

[44] Zhou C, He L, Yang N. Variations in the Ebinur Lake area caused by human activities and climatic changes. Mar Geol Quatern Geol. 2010;30:121–126. In Chinese with English abstract.

[45] Nesbitt HW, Markovics G. Chemical processes affecting alkalis and alkaline earths during continental weathering. Geochim Cosmochim Acta. 1980;44(11):1659–1666.

[46] Nesbitt HW. Mobility and fractionation of rare earth elements during weathering of a granodiorite. Nature. 1979; 279, 206–210.

[47] Schiff K, Weisberg SB. Iron as a reference element for determining trace metal enrichment in Southern California coastal shelf sediments. Mar Environ Res. 1999;48(2):161–176.

[48] Nesbitt H, Young G. Prediction of some weathering trends of plutonic and volcanic rocks based on thermodynamic and kinetic considerations. Geochim Cosmochim Ac. 1984;48(7):1523–1534.

[49] Hu S, Deng C, Appel E, Verosub K. Environmental magnetic studies of lacustrine sediments. Chin Sci Bull. 2002;47(7):613–616.

