# Supplementary material for: Geochemical Responses to Anthropogenic and Natural Influences in Ebinur Lake Sediments of Arid Northwest China
Source: PLoS One. 2016 May 13;11(5):e0155819. doi: 10.1371/journal.pone.0155819 (PMC4866693; doi:10.1371/journal.pone.0155819)
Supplement: S1 Table — (PDF) [file pone.0155819.s001.pdf]

S1 Table. The geochemical data of elemental compositions in Ebinur Lake sediments.

Depth (cm)	Al [a]	Ba [b]	Be [b]	Ca [a]	Co [b]	Cr [b]	Cu [b]	Fe [a]	K[a]	Li [b]	Mg [a]	Mn [b]	Na [a]	Ni [b]	P [b]	Pb [b]	Sr [b]	Ti [b]	V [b]	Zn [b]
1	39.78	291.97	1.24	77.35	7.22	35.26	18.76	19.83	14.07	35.17	21.15	411.45	42.61	19.72	441.00	13.91	696.09	2128.79	53.53	58.99
2	48.94	349.12	1.51	82.05	8.34	43.51	24.96	25.03	16.90	47.58	21.63	494.68	51.49	23.64	489.25	19.24	745.17	2136.81	65.48	75.79
3	50.07	364.70	1.70	74.13	8.34	43.94	20.29	24.17	17.53	42.47	18.70	517.33	33.77	26.17	608.20	19.83	628.03	2929.71	64.61	81.50
4	50.47	362.34	1.73	79.61	8.60	47.14	24.35	27.31	17.89	49.51	20.53	544.97	38.83	28.14	533.26	19.36	725.00	2741.58	69.19	79.43
5	47.92	340.23	1.58	91.52	8.42	44.35	23.99	26.28	17.22	51.58	21.93	537.52	41.23	28.29	452.11	17.76	943.27	2373.47	68.60	80.34
6	51.05	367.62	1.73	83.82	8.98	48.83	27.32	27.36	18.21	54.19	22.07	572.41	43.19	29.51	491.76	21.64	770.46	2583.78	72.25	82.62
7	51.45	362.74	1.71	82.26	9.11	50.32	27.83	28.95	18.35	54.74	22.92	572.73	42.26	30.07	489.34	21.49	797.20	2547.44	73.75	75.81
8	49.73	353.72	1.66	81.42	8.78	46.84	25.43	27.16	17.66	51.86	22.04	547.32	45.40	28.22	499.88	19.28	783.21	2567.30	70.92	64.37
9	50.80	379.53	1.71	81.85	9.12	45.80	22.93	25.92	17.80	48.97	20.24	551.48	36.80	27.18	532.54	18.89	745.76	2776.59	69.40	65.31
10	49.14	349.05	1.69	84.47	9.06	49.47	25.49	28.95	17.53	53.61	22.28	568.88	46.31	29.12	473.47	19.82	841.37	2539.35	71.58	68.95
11	49.96	348.49	1.58	86.47	9.71	47.84	28.42	27.88	17.61	52.25	23.63	564.51	36.97	27.36	479.31	19.74	845.83	2503.51	72.64	99.11
12	51.02	363.04	1.73	88.70	9.29	47.71	27.02	29.14	18.23	56.18	23.05	582.28	40.17	28.94	478.36	19.90	881.03	2554.72	75.60	87.87
13	50.70	395.41	1.71	87.50	9.32	49.62	27.55	28.92	18.19	56.17	22.40	582.34	42.51	29.45	483.86	20.09	880.00	2547.86	75.43	81.30
14	51.23	370.01	1.73	87.14	9.26	49.38	27.74	28.65	18.29	56.50	22.38	584.75	43.44	29.33	506.44	21.14	860.97	2601.03	74.52	93.96
15	51.46	369.91	1.72	86.36	9.30	48.68	28.69	28.80	18.41	55.67	22.63	573.42	41.25	29.93	500.18	21.64	858.38	2599.36	74.08	93.00
16	52.26	381.36	1.79	86.55	9.20	48.57	27.55	28.94	18.55	55.13	21.65	582.35	40.55	28.86	504.47	21.60	827.15	2658.62	74.19	96.56
17	50.93	369.57	1.69	91.58	9.45	47.97	27.51	27.77	17.84	52.36	22.04	567.61	38.12	27.57	497.63	20.18	937.02	2556.49	72.18	95.90
18	49.60	357.77	1.58	96.61	9.70	47.36	27.47	26.61	17.14	49.58	22.42	552.87	35.70	26.27	490.80	18.76	1046.88	2454.35	70.17	95.23
19	53.11	379.09	1.79	79.75	9.51	51.03	30.45	28.66	18.82	56.40	23.56	610.61	41.72	30.35	510.42	22.15	714.66	2611.90	78.00	85.43
20	52.54	372.95	1.79	85.62	9.28	50.36	30.57	29.97	18.65	58.10	23.23	606.77	42.00	29.92	506.48	20.26	846.13	2585.79	77.06	80.14
21	52.38	367.57	1.77	85.12	9.56	51.27	30.84	29.82	18.30	56.27	23.76	616.95	41.73	30.66	503.55	21.16	853.99	2579.56	77.35	87.63
22	52.18	374.65	1.79	83.91	9.80	50.23	29.38	29.78	18.87	55.89	23.75	613.35	39.17	30.48	525.18	21.20	824.83	2628.12	77.12	75.32
23	52.44	397.07	1.78	83.04	9.52	50.38	28.94	29.13	18.65	53.99	22.70	591.34	38.72	29.66	513.23	21.48	792.12	2652.70	75.07	78.67
24	52.70	389.76	1.74	82.58	9.19	47.29	25.02	27.67	18.19	52.02	20.78	596.77	32.22	28.23	557.17	20.73	744.85	2833.34	71.26	92.65
25	51.84	376.99	1.77	82.34	8.54	43.37	24.82	26.17	18.15	48.98	19.35	571.19	33.58	25.94	527.35	21.28	748.53	2677.02	66.43	91.84
26	49.83	350.11	1.67	95.96	8.56	45.12	25.74	26.24	17.54	50.98	19.06	574.61	36.38	26.36	503.62	21.01	947.49	2542.03	66.24	79.00
27	49.52	371.70	1.69	98.35	8.48	45.22	28.16	26.67	17.35	52.85	19.50	584.08	34.03	26.79	500.10	21.17	971.19	2508.05	67.57	74.73
28	50.02	359.41	1.67	97.38	8.43	43.53	27.78	26.17	17.58	50.47	18.65	566.01	33.47	26.32	491.57	19.91	980.03	2500.20	64.91	66.84
29	50.28	381.74	1.74	94.43	8.74	46.55	30.44	26.89	17.63	51.21	18.89	574.63	32.10	27.05	504.42	21.80	925.09	2563.07	68.00	73.95
30	49.85	366.75	1.77	92.68	8.20	40.58	20.56	24.05	17.75	44.22	16.52	530.54	29.42	24.90	525.06	18.79	890.32	2597.49	62.12	70.15
31	50.72	373.49	1.62	89.52	8.56	41.20	21.49	24.47	17.93	44.63	16.74	528.12	30.87	25.31	523.41	19.62	862.71	2629.71	62.20	73.62
32	53.07	373.94	1.77	87.74	9.23	47.02	27.11	28.95	18.27	54.79	19.26	627.89	31.10	28.59	512.06	21.91	800.62	2677.98	70.44	76.04
33	51.97	381.39	1.71	93.38	9.47	47.03	27.20	28.43	18.28	55.65	19.34	606.38	29.02	29.72	505.38	20.48	881.52	2667.07	73.80	87.17
34	51.92	374.24	1.72	94.53	9.15	47.66	27.16	28.38	17.98	55.25	19.13	591.41	30.26	29.39	516.86	20.35	897.71	2685.68	72.12	87.15
35	51.23	364.82	1.71	98.29	9.07	47.43	27.16	28.15	18.03	55.49	19.23	590.14	31.92	28.54	494.00	20.01	954.90	2590.66	72.57	64.94
36	51.52	404.45	1.73	89.05	9.27	46.87	24.08	26.73	17.86	49.75	17.51	567.71	29.87	26.78	536.92	21.01	815.95	2796.49	70.18	74.95
37	50.71	376.31	1.68	88.70	8.68	43.31	21.28	24.84	17.46	45.35	16.57	535.60	29.85	25.79	546.72	18.43	774.75	2793.33	65.94	78.83
38	50.61	357.77	1.75	104.33	9.55	48.09	27.29	29.43	17.88	59.17	20.54	604.78	33.70	28.95	477.90	22.41	1055.27	2493.13	75.79	83.39
39	50.87	374.45	1.75	89.76	9.35	45.89	24.63	26.82	17.64	50.14	17.82	570.95	29.22	27.66	538.17	20.08	810.91	2755.94	69.99	76.67
40	50.66	383.83	1.76	97.91	9.18	47.02	27.33	28.61	17.79	55.58	19.44	603.78	31.60	28.66	489.78	22.16	934.44	2588.91	74.38	74.99
41	50.23	358.81	1.74	102.35	9.53	48.64	27.59	28.89	17.51	55.97	19.39	615.03	30.62	28.47	535.15	20.41	998.40	2667.75	74.46	70.25
42	51.49	379.14	1.70	87.30	9.02	43.61	27.81	26.35	17.63	48.81	17.29	560.67	28.33	26.92	521.28	20.06	768.62	2770.38	68.27	72.19
43	51.87	379.80	1.79	91.28	9.22	47.75	26.04	28.21	18.02	53.71	18.48	581.34	27.99	28.77	528.12	20.91	851.84	2817.18	74.31	67.75
44	50.97	370.47	1.73	92.75	9.02	48.34	25.81	28.03	17.91	52.77	18.42	568.99	28.24	27.57	505.73	20.44	874.95	2681.47	72.56	63.05
45	52.33	410.04	1.79	92.15	9.18	47.87	26.36	28.57	18.09	53.04	18.56	568.64	27.60	27.87	522.15	21.62	839.73	2764.71	72.26	81.91
46	52.82	380.23	1.77	91.35	9.22	47.67	25.23	28.02	18.35	52.49	18.53	579.31	27.27	28.49	533.33	23.06	835.48	2837.43	73.47	99.75
47	51.37	370.17	1.76	97.30	9.24	47.39	29.33	28.76	18.03	57.37	20.17	587.20	30.88	28.61	490.09	22.22	958.21	2631.40	76.56	89.06
48	51.74	372.40	1.75	97.64	9.27	49.87	28.07	28.91	18.30	57.45	20.07	587.09	29.95	28.94	492.72	21.41	951.71	2623.14	76.97	92.32
49	52.35	374.80	1.78	100.75	9.56	50.09	30.34	30.65	18.52	59.17	20.93	604.84	29.58	30.60	495.87	23.01	1009.84	2603.18	78.32	87.18
50	52.16	390.66	1.78	98.06	9.16	49.08	29.78	30.03	18.34	58.52	20.20	595.29	29.11	29.65	473.54	21.64	964.39	2603.05	77.64	83.55

[a] the units for these elements is g kg^{-1};

[b] the units for these elements is mg kg^{-1}.
