# Supplementary material for: Geochemical Responses to Anthropogenic and Natural Influences in Ebinur Lake Sediments of Arid Northwest China
Source: PLoS One. 2016 May 13;11(5):e0155819. doi: 10.1371/journal.pone.0155819 (PMC4866693; doi:10.1371/journal.pone.0155819)
Supplement: S2 Table — (PDF) [file pone.0155819.s002.pdf]

S2 Table. Pearson correlation matrix for element concentration in Ebinur Lake sediments

		Al	Ba	Be	Ca	Co	Cr	Cu	Fe	K	Li	Mg	Mn	Na	Ni	P	Pb	Sr	Ti	V	Zn
Al	Pearson Correlation	1																			
	Sig. (2-tailed)																				
	N	50																			
Ba	Pearson Correlation	0.812[a]	1																		
	Sig. (2-tailed)	0																			
	N	50	50																		
Be	Pearson Correlation	0.893[a]	0.776[a]	1																	
	Sig. (2-tailed)	0	0																		
	N	50	50	50																	
Ca	Pearson Correlation	0.148	0.142	0.233	1																
	Sig. (2-tailed)	0.305	0.326	0.104																	
	N	50	50	50	50																
Co	Pearson Correlation	0.706[a]	0.518[a]	0.603[a]	0.263	1															
	Sig. (2-tailed)	0	0	0	0.065																
	N	50	50	50	50	50															
Cr	Pearson Correlation	0.699[a]	0.475[a]	0.668[a]	0.130	0.844[a]	1														
	Sig. (2-tailed)	0	0	0	0.368	0															
	N	50	50	50	50	50	50														
Cu	Pearson Correlation	0.544[a]	0.333[b]	0.497[a]	0.337[b]	0.690[a]	0.781[a]	1													
	Sig. (2-tailed)	0	0.018	0	0.017	0	0														
	N	50	50	50	50	50	50	50													
Fe	Pearson Correlation	0.735[a]	0.495[a]	0.728[a]	0.319[b]	0.836[a]	0.931[a]	0.822[a]	1												
	Sig. (2-tailed)	0	0	0	0.024	0	0	0													
	N	50	50	50	50	50	50	50	50												
K	Pearson Correlation	0.946[a]	0.729[a]	0.891[a]	0.094	0.702[a]	0.770[a]	0.589[a]	0.794[a]	1											
	Sig. (2-tailed)	0	0	0	0.515	0	0	0	0												
	N	50	50	50	50	50	50	50	50	50											
Li	Pearson Correlation	0.661[a]	0.416[a]	0.665[a]	0.435[a]	0.770[a]	0.877[a]	0.844[a]	0.961[a]	0.734[a]	1										
	Sig. (2-tailed)	0	0.003	0	0.002	0	0	0	0	0											
	N	50	50	50	50	50	50	50	50	50	50										
Mg	Pearson Correlation	-0.004	-0.234	-0.077	-0.339[b]	0.369[a]	0.534[a]	0.496[a]	0.421[a]	0.180	0.411[a]	1									
	Sig. (2-tailed)	0.976	0.102	0.596	0.016	0.008	0	0	0.002	0.210	0.003										
	N	50	50	50	50	50	50	50	50	50	50	50									
Mn	Pearson Correlation	0.814[a]	0.588[a]	0.822[a]	0.408[a]	0.796[a]	0.800[a]	0.762[a]	0.887[a]	0.814[a]	0.878[a]	0.177	1								
	Sig. (2-tailed)	0	0	0	0.003	0	0	0	0	0	0	0.218									
	N	50	50	50	50	50	50	50	50	50	50	50	50								
Na	Pearson Correlation	-0.307[b]	-0.440[a]	-0.353[b]	-0.558[a]	-0.121	0.126	0.078	-0.023	-0.129	-0.003	0.776[a]	-0.244	1							
	Sig. (2-tailed)	0.030	0.001	0.012	0	0.401	0.383	0.590	0.875	0.372	0.982	0	0.087								
	N	50	50	50	50	50	50	50	50	50	50	50	50	50							
Ni	Pearson Correlation	0.746[a]	0.495[a]	0.752[a]	0.165	0.784[a]	0.915[a]	0.730[a]	0.929[a]	0.842[a]	0.896[a]	0.425[a]	0.855[a]	0.023	1						
	Sig. (2-tailed)	0	0	0	0.252	0	0	0	0	0	0	0.002	0	0.874							
	N	50	50	50	50	50	50	50	50	50	50	50	50	50	50						
P	Pearson Correlation	0.425[a]	0.470[a]	0.412[a]	-0.261	0.058	-0.026	-0.295[b]	-0.112	0.286[b]	-0.237	-0.456[a]	0.099	-0.402[a]	-0.002	1					
	Sig. (2-tailed)	0.002	0.001	0.003	0.067	0.687	0.859	0.037	0.438	0.044	0.097	0.001	0.492	0.004	0.991						
	N	50	50	50	50	50	50	50	50	50	50	50	50	50	50	50					
Pb	Pearson Correlation	0.804[a]	0.650[a]	0.805[a]	0.338[b]	0.614[a]	0.688[a]	0.663[a]	0.749[a]	0.788[a]	0.737[a]	0.039	0.799[a]	-0.271	0.699[a]	0.177	1				
	Sig. (2-tailed)	0	0	0	0.016	0	0	0	0	0	0	0.789	0	0.057	0	0.218					
	N	50	50	50	50	50	50	50	50	50	50	50	50	50	50	50	50				
Sr	Pearson Correlation	0.082	0.015	0.156	0.928[a]	0.319[b]	0.230	0.441[a]	0.385[a]	0.105	0.496[a]	-0.045	0.400[a]	-0.298[b]	0.237	-0.439[a]	0.270	1			
	Sig. (2-tailed)	0.573	0.915	0.279	0	0.024	0.109	0.001	0.006	0.469	0	0.755	0.004	0.036	0.097	0.001	0.058				
	N	50	50	50	50	50	50	50	50	50	50	50	50	50	50	50	50	50			
Ti	Pearson Correlation	0.650[a]	0.683[a]	0.680[a]	-0.052	0.337[b]	0.259	-0.055	0.241	0.537[a]	0.116	-0.434[a]	0.401[a]	-0.592[a]	0.354[b]	0.811[a]	0.415[a]	-0.239	1		
	Sig. (2-tailed)	0	0	0	0.719	0.017	0.069	0.706	0.092	0	0.423	0.002	0.004	0	0.012	0	0.003	0.094			
	N	50	50	50	50	50	50	50	50	50	50	50	50	50	50	50	50	50	50		
V	Pearson Correlation	0.704[a]	0.485[a]	0.687[a]	0.262	0.868[a]	0.939[a]	0.791[a]	0.954[a]	0.769[a]	0.935[a]	0.470[a]	0.827[a]	-0.002	0.918[a]	-0.091	0.703[a]	0.322[b]	0.252	1	
	Sig. (2-tailed)	0	0	0	0.067	0	0	0	0	0	0	0.001	0	0.991	0	0.528	0	0.023	0.077		
	N	50	50	50	50	50	50	50	50	50	50	50	50	50	50	50	50	50	50	50	
Zn	Pearson Correlation	0.424[a]	0.247	0.256	0.051	0.472[a]	0.397[a]	0.354[b]	0.387[a]	0.420[a]	0.372[a]	0.339[b]	0.331[b]	0.010	0.361[a]	0.034	0.426[a]	0.130	0.141	0.433[a]	1
	Sig. (2-tailed)	0.002	0.084	0.072	0.724	0.001	0.004	0.012	0.005	0.002	0.008	0.016	0.019	0.944	0.010	0.814	0.002	0.370	0.330	0.002	
	N	50	50	50	50	50	50	50	50	50	50	50	50	50	50	50	50	50	50	50	50

[a] Correlation is significant at the 00.01 level (2-tailed)0.

[b] Correlation is significant at the 00.05 level (2-tailed)0.
